# A Complex Presentation of Esophageal Pseudodiverticulosis With Candida Albicans Esophagitis in a 68-Year-Old Female

**DOI:** 10.7759/cureus.55286

**Published:** 2024-02-29

**Authors:** Ismail Althunibat, Raed Atiyat, Byron Okwesili, Muhammad Hussain, Theodore Jr Dacosta

**Affiliations:** 1 Internal Medicine, Saint Michael's Medical Center, Newark, USA; 2 Gastroenterology, Saint Michael's Medical Center, Newark, USA; 3 Gastroenterology and Hepatology, Saint Michael's Medical Center, Newark, USA; 4 Gastroenterology and Hepatology, Saint Michael's Medical Center/New York Medical Center, Newark, USA

**Keywords:** candida, dysphagia, diverticulosis, esophageal, esophageal diverticulosis

## Abstract

Esophageal pseudodiverticulosis, a rare condition, involves small sac-like structures in the esophageal wall, stemming from dilated excretory ducts of submucosal glands. While uncommon, it can complicate *Candida albicans *esophagitis, a yeast infection linked to various clinical issues, including pseudodiverticula formation. This unique association underscores the importance of understanding its clinical implications and optimal management. In this case, a 68-year-old female sought medical attention for dysphagia and recurrent food impaction. The diagnostic journey revealed esophageal pseudodiverticulosis and *Candida albicans* esophagitis, emphasizing the complexity of esophageal disorders.

## Introduction

Esophageal pseudodiverticulosis (EPD) is a rare and benign condition characterized by outpouchings stemming from dilated excretory ducts of submucosal glands [1-5]. It is exceptionally uncommon, with a limited number of cases documented worldwide, estimated to be around 200. A comprehensive study analyzing 14,350 barium swallow esophagrams revealed its presence in only 21 patients, constituting a mere 0.15% of the total [2,3]. Esophageal candidiasis has been described in the pathogenesis of EPD. It is observed in approximately 26% of patients, but it could be related to stasis [4]. This unique association underscores the importance of putting *Candida* as one of the top differentials to be evaluated. In this case, we present a case of a 68-year-old female who was complaining of dysphagia and recurrent food impaction. Diagnostic evaluation revealed esophageal pseudodiverticulosis and *Candida albicans* esophagitis.

## Case presentation

**Figure 1:**
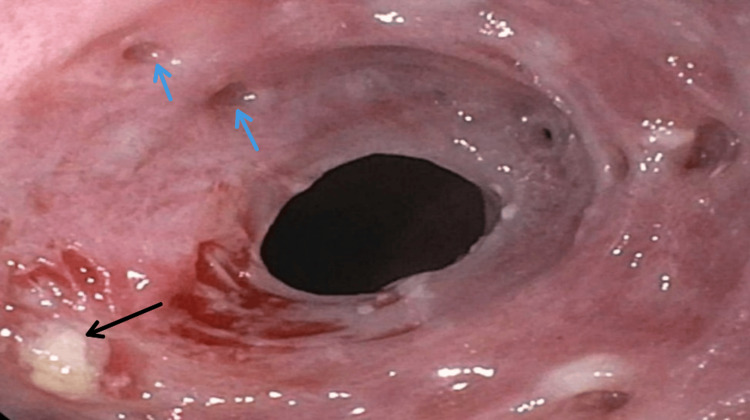
Severe esophagitis, extreme friability, white plaques (black arrow), and pseudodiverticula (blue arrows)

A 68-year-old African American female with hypertension, epilepsy, peripheral arterial disease, and hyperlipidemia presented with three years of progressive dysphagia and recurrent food impaction. The patient takes losartan, hydralazine, atorvastatin, aspirin, clopidogrel, and topiramate for the aforementioned medical conditions. A previous endoscopy two years before the presentation revealed benign esophageal stenosis and friable mucosa. She denied caustic agent ingestion, injury, or gastroesophageal reflux disease symptoms. She has a history of 28 pack-years of cigarette smoking. Despite ongoing dysphagia, she maintained a regular diet but avoided spicy food. An endoscopy was done and showed severe esophagitis, extreme friability, white plaques, pseudodiverticula, and benign stenosis at the upper esophageal sphincter, dilated during the procedure (Figure 1).

**Figure 2:**
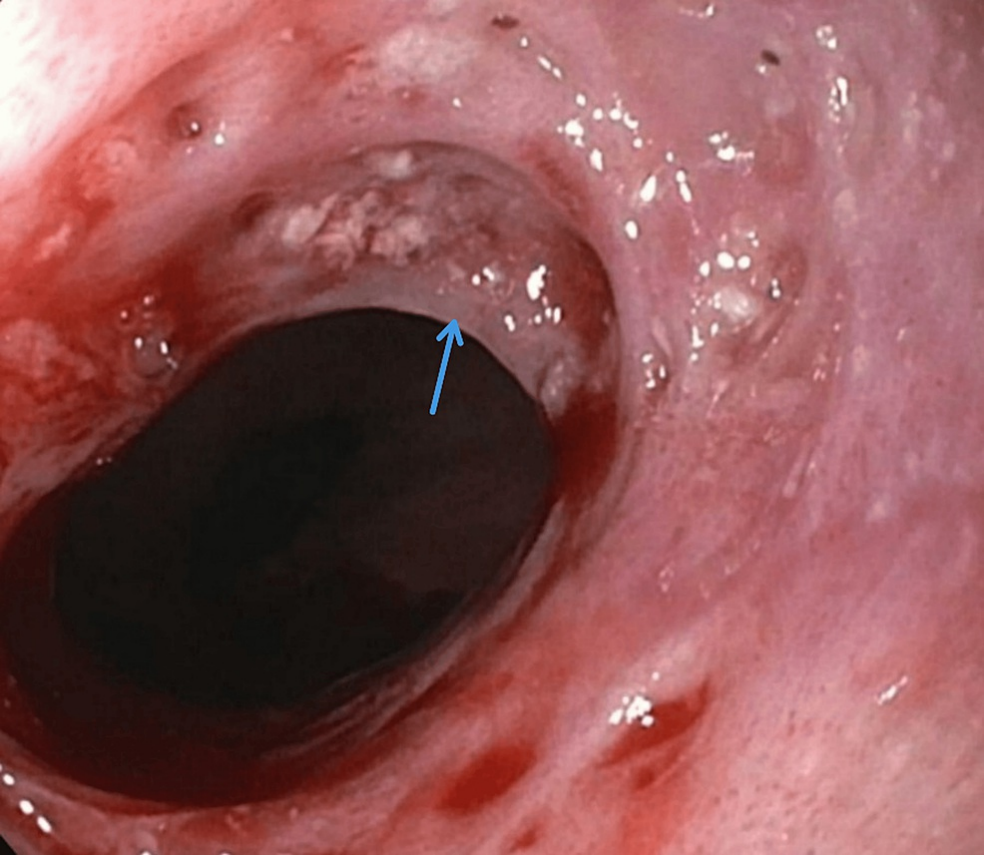
A high-grade Schatzki ring was found in the lower esophagus (blue arrow)

**Figure 3:**
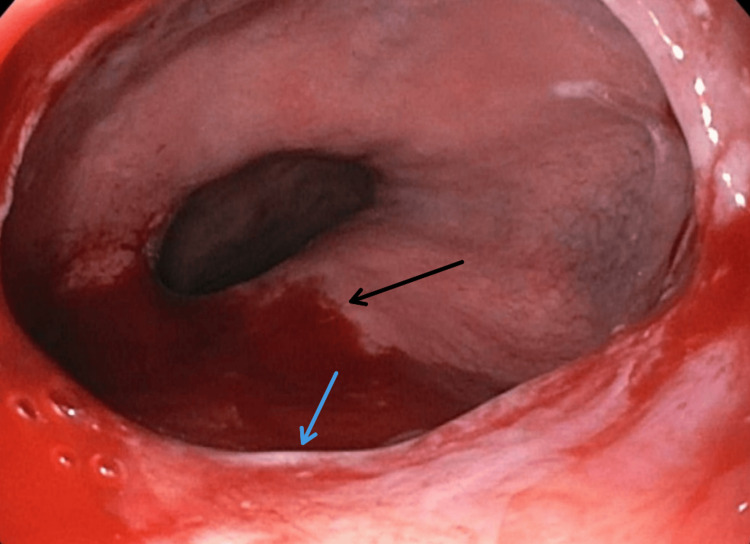
A high-grade Schatzki ring (blue arrow) along with diffuse moderate stomach inflammation (black arrow)

A high-grade Schatzki ring was found in the lower esophagus as well, along with diffuse moderate stomach inflammation (Figures 2, 3).

**Figure 4:**
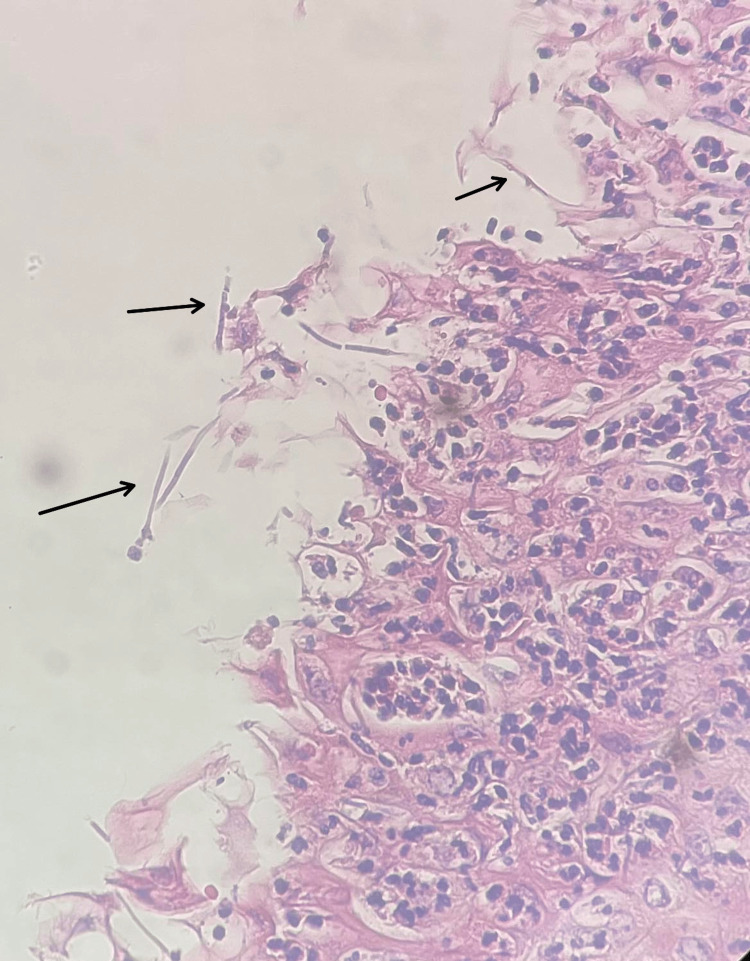
Squamous mucosa with severe inflammation and reactive changes with hyphae of Candida albicans (black arrows)

**Figure 5:**
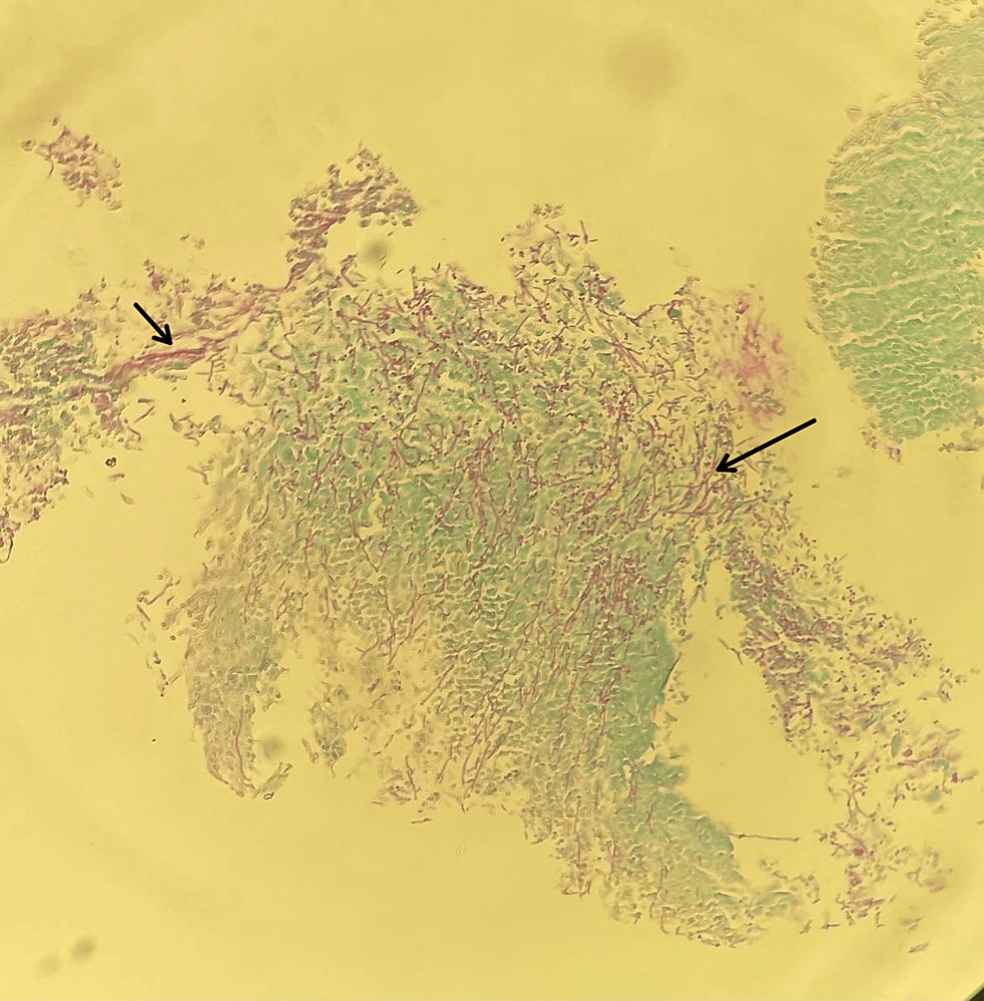
Candida species identified by PAS stain (black arrows) PAS stain: Periodic acid-Schiff stain.

Histopathology confirmed squamous mucosa with severe inflammation, reactive changes, and *Candida *organisms identified by periodic acid-Schiff (PAS) stain (Figures 4, 5).

After the diagnosis was established, HIV testing was done, which returned negative. The patient was initiated on fluconazole 200 mg daily for three weeks. Remarkably, the patient's symptoms exhibited a dramatic improvement, and she regained the ability to swallow both solids and liquids without difficulty. The patient refused to undergo a follow-up esophagogastroduodenoscopy (EGD) due to financial issues.

## Discussion

Esophageal diverticulosis is an uncommon condition characterized by the formation of pseudodiverticula in the wall of the esophagus. It is postulated that dilated excretory ducts of the submucosal glands due to chronic submucosal inflammation often predispose to the development of this condition by herniation of the mucosa through the sites of nerves and vessels supplying the area [1-5]. Diabetes mellitus, chronic alcohol consumption [6], esophageal candidiasis, and fungal infection are believed to be the most inflicted factors in the development of EPD with approximately 26% of EPD patients having concomitant esophageal candidiasis [4].

Dysphagia is the most common presenting symptom in such cases; however, odynophagia, chest pain, halitosis, and food impaction are also reported [7]. Chronic cases may progress into esophageal stenosis and severe dysphagia. Other less common complications reported include perforation with mediastinitis development and the formation of esophagobronchial fistula.

Evaluation starts with a proper analysis of the history of the symptoms. Barium esophagography can be the initial diagnostic test to be performed in suspected cases. Upper endoscopy can also be done to further evaluate the pseudodiverticula and obtain biopsies in cases of unclear underlying causes, as in the case we present [4,8].

In this present case, the patient's main complaint was dysphagia, which was progressive from solids to liquids. We performed upper endoscopy and confirmed the presence of esophageal diverticulosis in the setting of candidal esophagitis based on histologic features.

The treatment of EPD is based on the underlying cause. For cases with a background of subacute candidal esophagitis, antifungal medications (fluconazole) are the first line of treatment [9]; however, failure of treatment was reported in some cases that emphasizes the need for exploration for variable treatment modalities [5]. Esophageal dilatation should also be considered in cases with concomitant stenosis and stricture [10].

Based on that, a chronological evaluation for dysphagia should be implemented in every patient with symptoms of dysphagia. The diagnosis of esophageal pseudodiverticulosis needs a high index of suspicion, which can be only confirmed after proper investigations as mentioned above. The management of such cases can be difficult in some cases as mentioned in the literature; however, addressing the underlying cause should be done as an initial step, although there is no standard of care till now, which necessitates further studies and evaluations of similar cases.

## Conclusions

This case demonstrates the importance of keeping candida infection in mind in cases of dysphagia that do not respond to empiric and regular management. It also emphasizes the fact of association between candida esophagitis and the possible consequent pseudodiverticulosis formation in the esophagus. However, a multidisciplinary approach should be implemented in the approach for such cases. In addition, the rapid resolution of symptoms after fluconazole treatment in this case and the other cases mentioned in the literature puts antifungal medications at the top of modalities of treatment in such cases, despite the fact they are not always curative management, as some reported cases showed no improvement and even ultimately death. In conclusion, this case should encourage looking for alternative treatment modalities and addressing possible hidden factors that could play a role in the disease's outcomes and its responsiveness to treatment.
